# Evaluation of Piecewise Polynomial Equations for Two Types of Thermocouples

**DOI:** 10.3390/s131217084

**Published:** 2013-12-12

**Authors:** Andrew Chen, Chiachung Chen

**Affiliations:** 1 Department of Electrical Engineering, National Taiwan University, Taipei 10617, Taiwan; E-Mail: abcorchids@gmail.com; 2 Department of Bio-Industrial Mechantronics Engineering, National ChungHsing University, Taichung 40227, Taiwan

**Keywords:** piecewise polynomial equation, regression analysis, thermocouples

## Abstract

Thermocouples are the most frequently used sensors for temperature measurement because of their wide applicability, long-term stability and high reliability. However, one of the major utilization problems is the linearization of the transfer relation between temperature and output voltage of thermocouples. The linear calibration equation and its modules could be improved by using regression analysis to help solve this problem. In this study, two types of thermocouple and five temperature ranges were selected to evaluate the fitting agreement of different-order polynomial equations. Two quantitative criteria, the average of the absolute error values |*e*|_ave_ and the standard deviation of calibration equation e_std_, were used to evaluate the accuracy and precision of these calibrations equations. The optimal order of polynomial equations differed with the temperature range. The accuracy and precision of the calibration equation could be improved significantly with an adequate higher degree polynomial equation. The technique could be applied with hardware modules to serve as an intelligent sensor for temperature measurement.

## 1. Introduction

Temperature measurement is basic and important work in a variety of industries. Electrical temperature sensors included resistive temperature detectors, thermistors and thermocouples [1,2]. Because of their multiple advantages of low cost, robustness and easily standardization, thermocouples are the most frequently used sensors for temperature measurement. They can measure a wide range of temperatures and have long-term stability and high reliability [3,4]. However, the major problems of this sensor for signals conditioning are the cold junction compensation and linearization of the transfer relationship between temperature and output voltage [2,3].

Output voltage tables for various types of thermocouples list the output voltage corresponding to different temperatures [5]. The reference junction is fixed at 0 °C. The relation between output voltage and temperature is established as a higher order polynomial equation for each type thermocouple [1,3]. For T-type thermocouples, the relation equation is an 8th order polynomial equation for the temperature range from 0–400 °C. For practical applications, this calibration equation is expressed as an inverse equation. Temperature is recognized as the dependent variable and the output voltage serves as the independent variable.

Because these calibration equations are higher order polynomial equations, Sarma and Boruan [6] suggested that the whole temperature range can be divided into smaller ranges, with lower degree polynomial calibrations being used for each range [4], but the literature contains no reports of any applications of this method.

Hardware modules have been designed to linearize the non-linear signals with hardware linearization [7]. The curve of nonlinear signals was divided into several pieces. The relationship between input and output was assumed to be a linear equation. The thermocouple input signal for each piece was filtered, isolated, amplified and converted to an analog voltage output by a linear equation [6].

The theory of the calibration with piecewise linear regression has been discussed [8]. Several self-compensation methods were proposed to build reconfigurable measurement systems for designing intelligent sensors [9]. The thermistor output from 0 °C to 100 °C was selected to compare errors of the measurement system. However, the piecewise linear interpolation method had the largest errors for these methods.

Some generalized software techniques for linearisation transducers had been used for thermocouples [10,11]. However, their performances have seldom been reported. An increase in table size of the thermocouple output voltage could improve the accuracy, but is impractical for an electrical thermometer. More electronic circuits for linearization could enhance the accuracy. However, these circuits are affected by ambient temperature, electromagnetic, and radiofrequency interference [10,11]. A log-amplifier based circuit for linearizing thermocouple signals was described [12]. Three types of thermocouples were selected to compare simulation results. The maximum percentage nonlinearity error before and after linearization were reduced significantly. To design a higher precision industrial temperature measurement system, Sarma *et al.* [13] linearized the amplified thermo-emf of a K-type thermocouple with the least squares polynomial fitting technique. Four temperature ranges were selected. The parameters for linear and polynomial equations were estimated for receiving signals gained by the amplifier and the accuracy was better with polynomial equations than linear fitting.

Sarma and Boruan [6] developed a measurement system for a K-type thermocouple with analog-to-digital converter, amplifier reference junction and computer. The measurement temperature range was 0 °C to 200 °C. Two calibration equations, a 9th order polynomial and a linear model, were proposed by a least squares method. The accuracy was within ±0.08 °C at 100.2 °C standard temperature. The authors suggested that the precision could be improved with a higher order regression equation, but did not report their adequate regression model. Danisman *et al.* [14] designed a high precision temperature measurement system based on an artificial neural network for three types of thermocouples. A neural linearizer was used to compute the temperature from the output voltage of the thermocouples.

For determining the optimal order of polynomial equations for temperature measurement, data fitting ability and prediction performance are both important [15]. A higher order polynomial equation has higher values for the coefficient of determination (R^2^). However, the standard values of estimation could be increased with the loss of data freedom. A higher degree polynomial equation may be over-fitted and the predicted ability thus decreased [16]. Resistance-temperature calibration equations for a negative temperature coefficient (NTC) thermistor have been evaluated with a modern regression technique to show the importance of an adequate calibration equation [16]. The division of the whole measurement range into smaller temperature ranges was proposed [6]. These calibration equations could be transformed with the use of software and incorporated into an intelligent sensor.

In the previous studies, the curves of the relationship of temperature and output voltage were divided into many pieces. Each piece of these curves was assumed as a linear relationship, however, the residual plots of each piece still indicated nonlinear results [4,7,13]. The linear equation should not be the only choice for establishing of calibration equations. Least squares-based parabolic regression had been reported to determine the parameters of the calibration equation [17]. As the piece relationship between temperature and output voltage of a thermistor was assessed with the 4th order polynomial equation, the accuracy and precision could be improved significantly [16].

In this study, the data of output voltage for two types of thermocouple were used from the US National Institute of Standards and Technology (NIST) standard. Five temperature ranges were selected to evaluate their calibration polynomial equations, called piecewise polynomial equations. The parameters for these equations were estimated by the least squares technique. The fitting performance of these equations was evaluated by several statistical methods.

## 2. Calibration Equations

### 2.1. Calibration Equations

The inverse calibration equation was used to describe the relationship between temperature (T) and output voltage of thermocouples (mv). Because the output voltage at 0 °C for thermocouples is zero, the intercept is excluded in a polynomial equation:

(1) $$\text{T}={\text{c}}_{1}\text{mv}+{\text{c}}_{2}\text{m}{\text{v}}^{2}+\ldots \ldots .+{\text{c}}_{\text{k}}\text{m}{\text{v}}^{\text{k}}$$

where c_1_, c_2_ to c_k_ are constants.

### 2.2. Temperature-Voltage Data of Thermocouples

Table data for thermocouples [5] were selected to evaluate the fitting ability of the calibration in this study.

#### 2.2.1. Type of Thermocouples: T-Type and J-Type

Two-types of thermocouples were selected in this study for their popularity in industry. The method developed in this study could be used for other thermocouples. The J-type thermocouple is commonly used for higher temperature ranges. In this study, the type of thermocouple was selected to evaluate the improved performance by piecewise polynomial equation.

#### 2.2.2. Piecewise Range of Temperature

**Figure 1.**
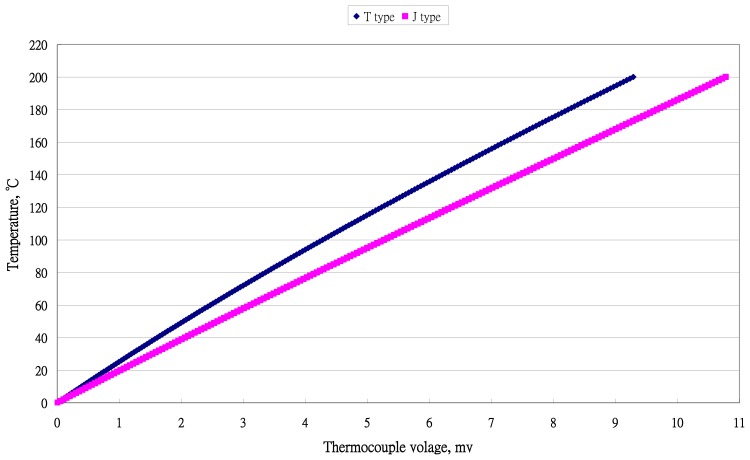
Distribution of temperature and output voltage of two types of thermocouples with temperature (0 to 200 °C).

**Figure 2.**
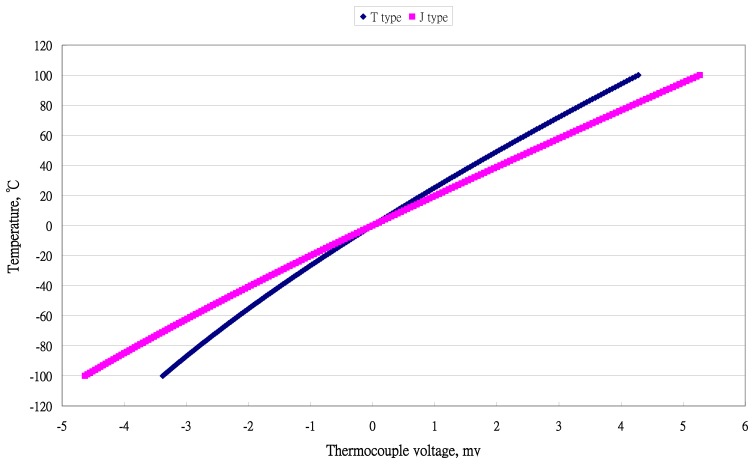
Distribution of temperature and output voltage of two types of thermocouples with temperature (−100 to 100 °C).

There were five ranges (a) 0∼100 °C; (b) 0∼200 °C; (c) −50∼50 °C; (d) −100∼0 °C; and (e) −100∼100 °C. They are the ranges for most living systems, included human beings. The distribution of temperature data for temperature *versus* voltage for two types of thermocouples are presented in Figures 1 and 2.

### 2.3. Data Analysis

Microsoft Excel 2003 was used to estimate the parameters of the different order polynomial equations. The *t* value of the highest order parameter was used to evaluate the optimal order of polynomial equations. If the order of polynomial equation is underestimated, the estimated parameters and variance will have a fixed bias. If the order of polynomial equation is overestimated, the variance increases and the bias of the prediction ability will inflate [6,15]. Residual plots were used as the qualitative criterion to evaluate the adequateness of models [15]. If the model is adequate for expressing the relationship between independent and dependent variables, the error distribution in residual plots is represented as horizontal bands. If the model is not adequate, the residual plots show a clear systematic pattern. The error was defined as follows:

(2) $$e_{i}=y_{i}-{\hat{y}}_{i}$$

where e_i_ is the error of calibration equation, y_i_ is the dependent variable and *y_i_* is the predicted values of the calibration equation.

Three statistics, e_max_, e_min_ and |*e*|_ave_ were used as quantitative criteria. The e_max_ is the maximum e_i_ value, e_min_ is the minimum e_i_ value and |*e*|_ave_ is the average of the absolute errors:

(3) $${\left|e\right|}_{\text{ave}}=\frac{\sum \left|e_{i}\right|}{n}$$

where |*e_i_*| is the absolute value of e_i_ and n is the number of data. The smaller of the |*e*|_ave_, the better the accuracy of the calibration equation.

The other criterion for uncertainty comparing of calibration equations is precision. The precision performance could be calculated from the standard deviation of the calibration equation [18]:

(4) $$e_{\text{std}}={\left(\frac{\sum {e_{i}}^{2}}{n-1}\right)}^{0.5}$$

## 3. Evaluation of Calibration Equations of Thermocouples

### 3.1. T-Type Thermocouple

The estimated parameters of calibration equations for five temperature ranges are listed in Table 1. The quantitative criteria for these calibration equations are listed in Tables 2 and 3.

#### 3.1.1. Range 0∼100 °C

**Figure 3.**
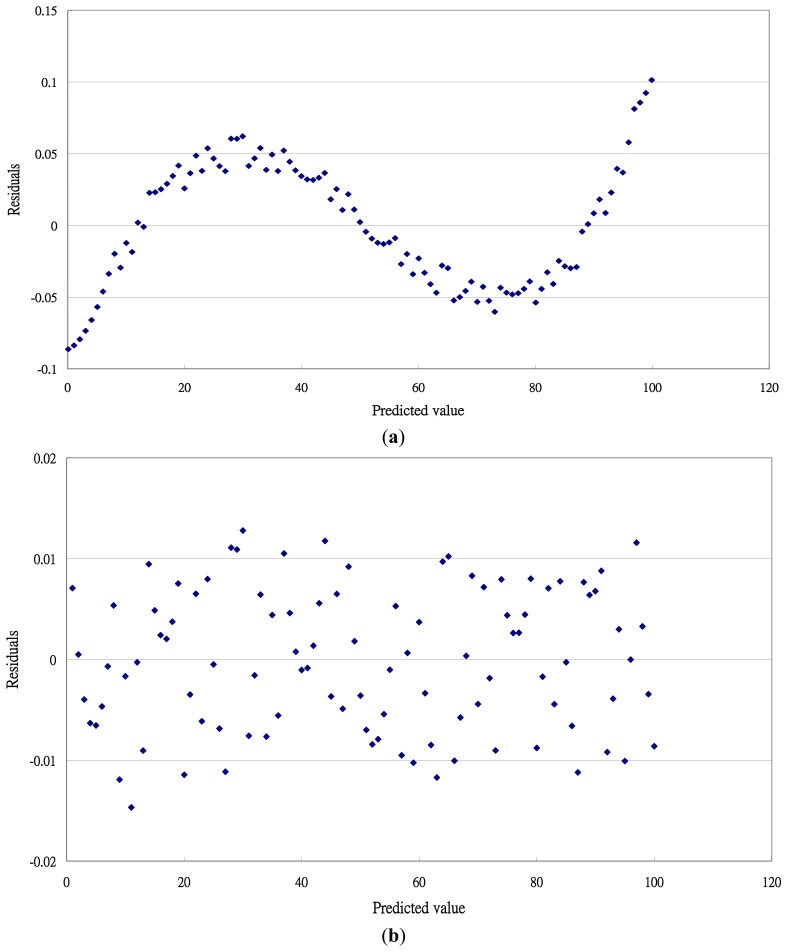

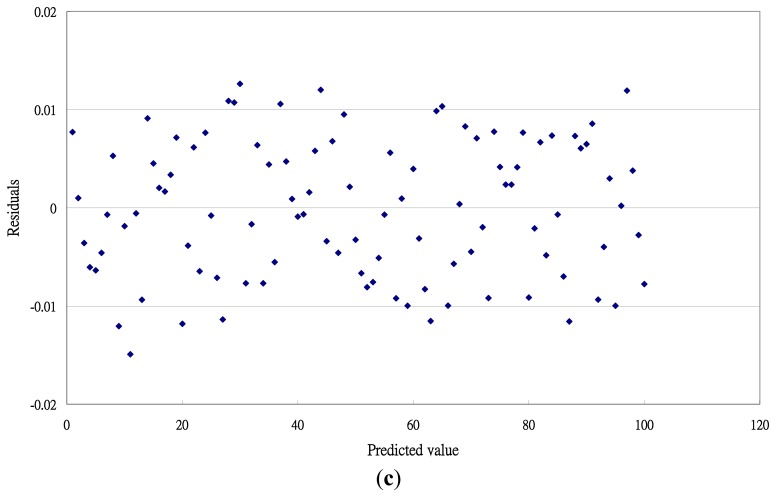
Residual plots of polynomial calibration equations for T-type thermocouples with temperature 0 to 100 °C. (**a**) 2nd order polynomial equation; (**b**) 3rd order polynomial equation; (**c**) 4th order polynomial equation.

The 2nd order polynomial equation produced a clear systematic pattern of residual plots (Figure 3a). The 3rd and 4th order polynomial equations produced a random distribution on residual plots (Figure 3b and c). Thus, the 2nd polynomial equation was not adequate because of systematic errors were found over the temperature ranges.

The |*e*|_ave_ value represents the accuracy of the calibration equation. From Table 2, the 2nd order polynomial equation had the largest value for e_max_, e_min_ and |*e*|_ave_. The |*e*|_ave_ values for the 3rd and 4th order polynomial equations did not differ substantially: 0.00681306 and 0.00676768, respectively.

The e_std_ value represents precision of the calibration equation. The e_std_ values for the 2nd, 3rd and 4th order polynomial equations were 0.05325660, 0.00840050 and 0.00824098, respectively (Table 3). The reduction in e_std_ values between 2nd and 3rd order polynomial equation was about 1/6.5 but that between 3rd and 4th order polynomial equations was not substantial. The increase in the 4th order (c_4_x^4^) of the calibration equation had only a marginal effect on improving performance. The adequate calibration equation for the T-type thermocouple for temperature 0 to 100 °C is as follows:

(5) $$T=25.86464325mv-0.69457635mv^{2}+0.026133029mv^{3}$$

#### 3.1.2. Range 0∼200 °C

The 2nd and 3rd order polynomial equations produced a systematic residual pattern and 4th and 5th order polynomial equations revealed a uniform distribution. All residual figures were showed in Supplement Figures A.

The |*e*|_ave_ values for the 2nd, 3rd, 4th and 5th order polynomial equations were 0.36507503, 0.03911083, 0.00718054 and 0.00680437, respectively (Table 2). Therefore, the 4th and 5th degree equations had the best accuracy.

The e_std_ values for the above four equations were 0.41512994, 0.04580101, 0.00940073 and 0.00860020, respectively (Table 3). Comparing 3rd degree equation with 4th degree equation, the reduction in e_std_ value between 3rd and 4th order equation was approximately 1/5. Comparing with the 4th order equation, the contribution of the 5th order equation was substantial. The reduction in precision was limited. Therefore, the adequate equation for the T-type thermocouple for temperature 0 to 200 °C is as follows:

(6) $$T=25.90205757mv-0.73340079mv^{2}+0.037584526mv^{3}-9.9772501\times 10^{-4}mv^{4}$$

#### 3.1.3. Range −50∼50 °C

This temperature range included the activity environment for the most biological system. The residual plots of the 2nd, 3rd and 4th order calibration equations are presented in Supplement Figure B. Only the 4th order equation showed a random distribution in residual plot. The 4th order polynomial had the smallest |*e*|_ave_ and e_std_ values (Tables 2 and 3). The following equation was considered as adequate:

(7) $$T=25.84551540mv-0.70994624mv^{2}+0.074689216mv^{3}-0.018167033mv^{4}$$

#### 3.1.4. Range −100∼0 °C

Only the 4th order polynomial equation had a uniform distribution on residual plots (data not shown) and the smallest value of |*e*|_ave_ and e_std_ (Tables 2 and 3). This following equation was considered adequate:

(8) $$T=25.77505075mv-0.83058517mv^{2}+0.026571395mv^{3}-0.018427604mv^{4}$$

#### 3.1.5. Range −100∼100 °C

The shape of the data distribution between temperature and thermocouple output voltage is a nonlinear curve. Only a higher order polynomial equation could produce a uniform distribution on residual plot (data not shown). The adequate calibration equation was a 6th order polynomial equation and showed as follows:

(9) $$T=25.85453185mv-0.72787713mv^{2}+0.067478989mv^{3}-1.2651926\times 10^{-2}mv^{4}+6.0999501\times 10^{-4}mv^{5}-1.3091201\times 10^{-4}mv^{6}$$

The |*e*|_ave_ value represents the accuracy and the e_std_ value was used to assess the precision of these equations. By the selection of the adequate polynomial calibration equations, the |*e*|_ave_ was < 0.009 °C and the e_std_ value was < 0.012 °C for the T-type thermocouple.

### 3.2. J-Type Thermocouple

The estimated parameters for calibration equations for five temperature ranges are listed in Table 4 and the quantitative criteria are listed in Tables 5 and 6.

All datasets for different temperature ranges were evaluated by regression analysis. The residual plots were used to evaluate the adequateness of models. The |*e*|_ave_ and e_std_ values were used to assess accuracy and precision. The adequate equations for different temperature ranges are listed as follows:

(1) 1.0∼100 °C
  (10) $$T=19.82859586mv-0.214978825mv^{2}+0.01024941mv^{3}$$
(2) 0∼200 °C
  (11) $$T=19.8289561mv-0.21979759mv^{2}+0.012147965mv^{3}-3.0010410\times 10^{-4}mv^{4}$$
(3) −50∼50 °C
  (12) $$T=19.84610586mv-0.23898495.mv^{2}+0.020179476mv^{3}-1.2941520\times 10^{-3}mv^{4}$$
(4) −100∼0 °C
  (13) $$T=19.85185466mv-0.225995822mv^{2}+0.030341877mv^{3}-2.5509630\times 10^{-3}mv^{4}+6.2928705\times 10^{-4}mv^{5}$$
(5) −100∼100 °C
  (14) $$T=19.84959392mv-0.238449137mv^{2}+0.018639399mv^{3}-1.4776301\times 10^{-3}mv^{4}+1.5145009\times 10^{-4}mv^{5}-1.2754301\times 10^{-5}mv^{6}$$

With the selection of the adequate polynomial calibration equations, the |*e*|_ave_ was <0.005 °C and The e_std_ value was <0.008 °C for the J-type thermocouple. The |*e*|_ave_ value presented the accuracy and the e_std_ value showed the precision of these equations. These numeric values indicated the performance improvement for this type thermocouple using in the special temperature range.

Now, the development of microprocessor systems is rapid and the price is dwindling. The nonlinear characteristics of sensing element could be improved by software package techniques. The calculation of the higher order polynomial equation could be treated as rapidly and accurately as linear equations. In this study, the orders of their polynomial equations for adequate calibration equations were lower than that of the NIST Standards. The accuracy and precision of these equations were improved significantly compared to that of a linear equation. They could be adapted to microprocessor systems to enhance the measurement performance of different types of thermocouples. The suggestion of the application of these polynomial calibration equations are as follows:

- The analog mv output of the thermocouple is amplified to voltage.
- The voltage signal is digitized by A/D converter.
- The function of the A/D converter is controlled by a microcomputer.
- The software for these calibrations is embedded in the flash ROM of the microcomputer.
- The true temperature then is computed by its adequate polynomial calibration equation.
- The true temperature could be display in a LCD or send to a PC via RS232 for data display or send to temperature controller.

## 4. Conclusions

Thermocouples are the most frequently used sensors for temperature measurement. However, linearizing the transfer relationship between temperature and output voltage is one of their major problems. In this study, two types of thermocouple with five temperature ranges were selected to evaluate the fitting agreement of different order polynomial equations to help solve this problem. The estimated parameters were established by regression analysis techniques. Two quantitative criteria, |*e*|_ave_ and e_std_ were used to evaluate the accuracy and precision of these calibrations equations. Residual plots were applied to justify the adequateness of these models.

The adequate order of polynomial calibration equation was affected by the temperature range. The 3rd order polynomial equation was adequate for the 0 to 100 °C temperature range and the higher 6th order polynomial equation was adequate for the −100 °C to 100 °C range.

The |*e*|_ave_ value represents the accuracy of these equations. The e_std_ value was used to assess the precision of equations. With the adequate polynomial calibration equation, the |*e*|_ave_ was <0.009 °C for the T-type thermocouple and <0.005 °C for the J-type thermocouple. The numeric value of e_std_ was <0.012 °C for the T-type thermocouple and <0.008 °C for the J-type thermocouple.

These polynomial calibration equations are easy to be written as software and be incorporated into an IC circuit as calculated equations. The measured thermocouple output could be transformed into the temperature easily and accurately. The technique could be applied with hard modules to serve as intelligent sensors. The regression analysis technique and criteria for comparison used in this study could be applied to evaluate adequate calibration equations for other thermocouples with different temperature ranges. The piecewise polynomial equation could be established to meet the requirement temperature range for practical applications.

## Figures and Tables

**Table 1.** Estimated parameters for several polynomial equations for T-type thermocouples by temperature range.

**Equation**	**bi**	**0–100°C**	**0–200°C**	−**50–50°C**	−**100–0°C**	−**100–100°C**
2nd order	b_1_	25.67471979	25.07879340	26.00810515	−25.22117110	26.44647267
	b_2_	−0.54576494	−0.39314346	−0.74516432	−1.26190490	−0.78530785
3rd order	b_1_	25.86464325	25.76378439	25.85804386	−25.90849949	25.91435043
	b_2_	−0.69457635	−0.64264925	−0.75808252	−0.59839052	−0.83435521
	b_3_	0.026133029	0.020317674	0.066304477	−0.14489758	0.061098417
4th order	b_1_	25.84962602	25.09020576	25.84551540	−25.77505075	25.81912460
	b_2_	−0.673394463	−0.73340079	−0.70994624	−0.830585167	−0.74867280
	b_2_	0.017448349	0.037584526	0.074689216	−0.026571395	0.077691433
	b_4_	−1.082962 × 10^−3^	−9.9772501 × 10^−4^	−0.018167033	−0.018427604	−8.8817640 × 10^−3^
5th order	b_1_		25.88262726			25.86505358
	b_2_		−0.71357086			−0.73577069
	b_2_		0.031114204			0.062941133
	b_4_		−1.5600801 × 10^−4^			−0.010532441
	b_5_		3.7937780 × 10^−5^			9.7149801 × 10^−4^
6th order	b_1_					25.85453185
	b_2_					−0.72787713
	b_2_					0.067478989
	b_4_					−0.012651926
	b_5_					6.0999501 × 10^−^**^4^**
	b_6_					−1.3091201 × 10^−^**^4^**

**Table 2.** Criteria for evaluating of polynomial equations for T-type thermocouples by temperature range. Sacle equation to same font size as table.

**Equation**	**Criteria**	**0–100°C**	**0–200°C**	−**50–50°C**	−**100–0°C**	−**100–100°C**
2nd order	e_min_	−0.07447137	0.49823066	0.225686384	−0.36968565	−1.67100581
	e_max_	0.13074332	0.98345135	0.13642422	0.18487408	1.21440524
	|*e*|_ave_	0.04592460	0.36507503	0.06717221	0.12630630	0.48771413
3th order	e_min_	−0.02072832	0.13412573	0.06409870	−0.04361028	−0.55185176
	e_max_	0.01471193	0.07282170	0.04472070	0.03208275	0.285123028
	|*e*|_ave_	0.00681306	0.03911083	0.02223685	0.01384381	0.150423885
4th order	e_min_	−0.01753270	0.03052425	0.02023304	−0.01507971	−0.07094427
	e_max_	0.01501541	0.19169659	0.02069277	0.01633248	0.11870578
	|*e*|_ave_	0.00676768	0.00718054	0.00763593	0.00663725	0.027618094
5th order	e_min_		0.02534386			−0.027957131
	e_max_		0.01656904			0.03741114
	|*e*|_ave_		0.00680437			0.01217997
6th order	e_min_					-0.02814230
	e_max_					0.02771649
	|*e*|_ave_					0.00986177

**Table 3.** Measurement precision of polynomial equations for T-type thermocouples by temperature range.

**Equation**	**0–100°C**	**0–200°C**	−**50–50°C**	−**100–0°C**	−**100–100°C**
2nd order	0.05325659	0.41512994	0.079658494	0.14552443	0.57348457
3rd order	0.00840050	0.04580101	0.026619274	0.01675441	0.18449760
4th order	0.00824098	0.00940073	0.009181103	0.00794493	0.03658164
5th order		0.00860020			0.01527604
6th order					0.01228220

**Table 4.** Estimated parameters for several polynomial equations for J-type thermocouples by temperature range.

**Equation**	**bi**	**0–100°C**	**0–200°C**	−**50–50°C**	−**100–0°C**	−**100–100°C**
2nd order	b_1_	19.71440273	19.45794480	19.92321865	19.53896507	20.15400867
	b_2_	−0.14280031	−0.08790950	−0.24205613	−0.42806222	−0.25366061
3rd order	b_1_	19.82859586	19.76305984	19.84718826	19.91333650	19.85023043
	b_2_	−0.21497883	−0.18247776	−0.24479520	−0.16293062	−0.26518721
	b_3_	0.01024941	0.006582481	0.019753541	0.042391433	0.02046607
4th order	b_1_	19.84344081	19.82989561	19.84610586	19.83020340	19.82940836
	b_2_	−0.23187152	−0.21979759	−0.23898495	−0.26902673	−0.23785577
	b_3_	0.01584881	0.012647965	0.020179476	2.9937101 × 10^−3^	0.02258792
	b_4_	−5.6514610 × 10^−4^	−3.0010410 × 10^−3^	−1.2941520 × 10^−3^	−4.5105150 × 10^−3^	−1.5837460 × 10^−3^
5th order	b_1_				19.85185466	19.84739765
	b_2_				−0.22599582	−0.23586796
	b_3_				0.030341877	0.019194036
	b_4_				−2.5509630 × 10^−3^	−1.7327180 × 10^−3^
	b_5_				6.2928705 × 10^−4^	1.2591410 × 10^−4^
6th order	b_1_					19.84959392
	b_2_					−0.23844914
	b_3_					0.018639399
	b_4_					−1.4776299 × 10^−3^
	b_5_					1.5145010 × 10^−4^
	b_6_					−1.2754301 × 10^−5^

**Table 5.** Criteria for evaluating of polynomial equations for J-type thermocouples by temperature range.

**Equation**	**Criteria**	**0–100°C**	**0–200°C**	−**50–50°C**	−**100–0°C**	−**100–100°C**
2nd order	e_min_	0.05557809	0.24933012	0.13616157	−0.28775249	−1.18173165
	e_max_	0.08928586	0.47674018	0.11595334	0.14466809	0.85074564
	|*e*|_ave_	0.03400235	0.18601820	0.04196053	0.09499065	0.33772693
3rd order	e_min_	0.01317127	0.068261782	0.02101240	−0.04169692	−0.30645416
	e_max_	0.01187325	0.046436731	0.01547939	0.02779399	0.14204194
	|*e*|_ave_	0.00481871	0.021161537	0.00574589	0.01190433	0.07435890
4th order	e_min_	0.01009384	0.013460643	0.01074795	−0.01449671	-0.06051327
	e_max_	0.00925146	0.013254812	0.00786120	0.01273097	0.03879727
	|*e*|_ave_	0.00429203	0.004711247	0.00438609	0.00532295	0.01359488
5th order	e_min_				−0.01239944	−0.02524355
	e_max_				0.01075262	0.01869201
	|*e*|_ave_				0.00507465	0.00580843
6th order	e_min_					−0.01393513
	e_max_					0.01228582
	|*e*|_ave_					0.00482716

**Table 6.** Measurement precision of polynomial equations for J-type thermocouples by temperature range.

**Equation**	**0–100°C**	**0–200°C**	−**50–50°C**	−**100–0°C**	−**100–100°C**
2^nd^ order	0.03903280	0.21139799	0.049680	0.10986868	0.39874169
3^rd^ order	0.00585086	0.02519590	0.007350308	0.01429016	0.09409667
4^th^ order	0.00522916	0.00582213	0.005281434	0.00641780	0.016754189
5^th^ order		0.00537354		0.00612658	0.00723238
6^th^ order					0.00581152
